# A Social Network Approach to the Estimation of Perceived Quality of Health Care

**DOI:** 10.2174/1874434601711010219

**Published:** 2017-10-31

**Authors:** Giulia Carletti, Nicola Soriani, Martina Mattiazzi, Dario Gregori

**Affiliations:** 1 Department of Radiotherapy and Molecular Medicine, Istituto Oncologico Veneto, Padova, Italy; 2 Unit of Biostatistics, Epidemiology and Public Health, Department of Cardiology, Thoracic and Vascular Sciences, University of Padova, Padova, Italy; 3 Customer Relation Office, Istituto Oncologico Veneto, Padova, Italy; 4 Unit of Biostatistics, Epidemiology and Public Health, Department of Cardiology, Thoracic and Vascular Sciences, University of Padova, Padova, Italy

**Keywords:** Perceived Quality of Care, Social Networks, Patient satisfaction, Network Scale-up Method, Customer Relations Office, Leave-one out

## Abstract

**Background:**

Measuring service quality aids health care providers to recognize specific and unmet needs of patients. Nevertheless, perceived quality of health care services (PQC) is often investigated with inadequate techniques which may lead to biased results.

**Objective:**

The aim of the present study is to develop a proof-of-concept for estimating the PQC using the scale-up estimator, with reference to a concrete assessment in patients of a major Oncology Hospital in Veneto (IOV). Results have then been compared with those collected by the Customer Relations Office (CRO) after the annual survey conducted with traditional questionnaire based techniques.

**Material and Methods:**

Seven hundred and eighty-three sets consisting of two questionnaires were handed out to IOV patients between 26 and 28 November 2012. The first questionnaire was the CRO annual one composed by 15 direct questions about the perception of quality satisfaction rate using a Likert scale. The second questionnaire was the scale-up (NSUM) one, composed by 20 indirect questions, 5 of which were reproducing the main target of CRO for estimating PQC.

**Results:**

The comparisons made over 299 sets of questionnaires showed differences between the two techniques. Network Scale-Up Method (NSUM) questionnaire seems to be able to produce lower estimates of PQC with respect to the CRO annual questionnaire. In some cases, the NSUM showed dissatisfaction rates which are 20-fold higher respect to CRO.

**Conclusion:**

NSUM could be a promising method for assessing the perceived quality of care.

## 1 INTRODUCTION

The quality of services has become an increasingly investigated field in the healthcare systems of both developed and developing countries [1]. There is a growing consensus that this should not be limited to objective outcomes of care, but that shall also include patient satisfaction as an important parameter and as an indicator of care quality. Its assessment provides a useful feed-back to both the medical staff and to the general personnel [2-4]. Perceived Quality of Care (PQC) is considered a key factor in ranking health care services and it is considered as a potential source of sustainable competitive advantage. In this sense, its understanding, measurement, and improvement are representing an important although challenging step for the entire health services organization [5]. This is also true in systems where hospitals are competing in providing services, whose PQC can be used as a decisive distinction for building a unique advantage, difficult for rivals to follow or copy, determining patients’ preference for choosing a hospital, as well as in satisfying and keeping customers, sustaining their loyalty to the hospital [5]. Providing patients with the services matching their needs and expectations are crucial for the existence and success of the organization in the competitive background of health care. Further on this, Parasuraman and colleagues [6] defined the perceived quality of service as the results of consumers’ juxtaposition of expected service with perception of actual efficiency of the service [7, 8].

However, methodological issues often neglect the possibility of a sound and unbiased assessment of it [9, 10]. The assessment of PQC is commonly performed via self-administered questionnaires, distributed by nurses at the end of the hospital stay. Such assessment may be affected by several factors, like above all (*i*) patients’ health conditions, (*ii*) self-selection, consisting in a systematic non-disposal to participate to PQC surveys, (*iii*) the facility services used by patients [3, 9, 10] and finally some methodological aspects as (*iv*) the distribution of questionnaire itself, which can heavily influence the compilation and the reliability of answers provided [9, 11, 12].

In addition, problems also arise at the time of interpreting results, in the sense that the objective interpretation of the complex features of perceived quality is very hard to achieve, due to both the lack of neutrality involved in the commonly used surveys investigating service quality [13, 14] and the feed-backs, as reported by patients, which may also include personal evaluations, undermining the objectivity of the investigator [14-16].

Another essential aspect, which may influence PQC assessment is that patients may fear a rebound and are afraid that answers provided may affect the quality of their future treatment, therefore are not likely to provide completely true answers, most likely ending up in an overestimation of the perceived quality of the care [17].

In this view, a potentially fruitful approach could be represented by the usage of indirect assessment techniques, which, instead of being targeted to the individual respondents’ status, are focused on the knowledge they potentially have on other people with whom they have been in contact.

A tentative research direction in this sense is represented by those surveys which focus on the indirect concept of “recommendation” to others of the given hospital or health care facility [18]. Elaborating on the concept of “others” and putting it in the more formal context of social networks, a particularly sensitive estimator is that based on scaling up techniques, where the problem is re-parameterized in terms of assessing the size of a hidden subpopulation of unknown size, in our context: the number of people un-satisfied by the service received. Bernard [19-22] developed this concept proposing the Network Scale Up Methods (NSUM), which, after being first proposed in 1989, has been applied to several fields of investigation, like HIV+, injuries or social themes, including security against terrorism [23], showing a strong capability of overcoming the usual traps of classical techniques [24] with a wide potential of applicability in public health research [25].

The aim of the present study is to develop a proof-of-concept for estimating the perception of quality of health care using the scale-up estimator, with reference to a concrete assessment in patients of a major Oncology Hospital of Padova in Veneto. Results will be then be compared with those collected by the Customer Relations Office after the annual survey conducted with traditional direct questionnaire based techniques.

## 2 METHODS

This study was conducted between 26 and 28 November 2012 in a major Oncology Hospital (IOV) of Padova in Veneto (Italy) in conjunction with the annual PQC assessment, conducted by the IOV Customer Relations Office (CRO – *Ufficio Relazioni con il Pubblico*). The research was conducted within 13 patient wards: Imaging, Radiology and Pathology; Center Unique Reservation (CUP); Radiotherapy and Nuclear medicine; Clinical oncology; Surgical oncology; Diagnostic and Operative endoscopy; Breast surgery; Anesthesiology; Melanoma and soft tissue tumors; Cardiology; Pharmacy; Psycho-oncology; Immunology and Molecular Oncology. Patients surveyed for the present study include IOV patients diagnosed with cancer or who have already received treatment and are able to provide answer independently. Terminally ill patients suffering from alterations in cognitive functions and aged patients not benefiting from the support of caregivers have not been surveyed.

A set of two questionnaires was administered to each patient: the first was the official CRO survey of PQC, the second the NSUM. Demographic and basic health characteristics were shared in the two forms.

Each IOV Operating Unit (OU) received a number of sets proportional to the number of patients daily admitted to it during the three-day period when the CRO assessment was conducted. Patients were required to fill out the questionnaires without supervision. Questionnaires were returned anonymously in sealed envelopes.

### 2.1 CRO-PQC Questionnaire

The CRO-PQC questionnaire covers four aspects of the PQC, (*i*) the patient’s feed-backs on personnel, (*ii*) the waiting time, (*iii*) the comfort of staying in the hospital and (*iv*) the overall feed-back on the structure.

Originally, the questionnaire included only 10 questions that were later integrated, up to 15, with further questions aiming at investigating quality standards such as volunteers, privacy, information transparency and the criteria leading consumers to the choice of the Institute. A Likert satisfaction scale with four potential answers to choose from was developed. The first four questions required the patient’s personal information, while the rest investigated the quality satisfaction rate on a Likert scale from “good” to “inadequate”. Questions on waiting time refer to regional standards, as provided by the local health authorities. In this domain, two questions examined waiting time for medical consultation after scheduling an appointment, with a satisfaction scale rating from “short” to “very long”, and between scheduled appointment and consultation at the Institute, where the choice ranged from “less than 15 minutes” to “more than 60 minutes”.

### 2.2 NSUM Questionnaire

In the present study, we developed a survey making use of the scale-up method, which gathers information through indirect questions to each IOV patient individually. The basic idea of the scale-up method is that the mean number of people known in a subpopulation (*e.g.* people owning a specific car) is linearly proportional to the size of the corresponding population. Interviewees are not asked directly about problems, but are required to give an estimate of the number of people they know in each sub-population included in the survey. Then, such figures are used to estimate the social network size of each person, which eventually turns out to be used to re-proportionate to the whole population the answers provided with reference to the target populations (*e.g.* people dissatisfied with the service).

Questions were introduced by in the following way “how many people do you know …?”. The definition of “knowing” was the one adopted in Snidero *et al.* [24], *i.e.* “*mutually recognize each other by sight or name, can be contacted, and had a contact within the last two years, either in person, by phone or mail*”.

To produce the NSUM questionnaire, we firstly selected the known population sizes published on the Central Statistics Institute website [26]. Subsequently, we produced 29 questions for the pilot questionnaire based on the data collected (details on the sub-populations used are provided in the supplemental Table **A1**).

A first draft of the questionnaire with 29 general questions applying to a known population size was developed. After that, the pilot questionnaire was administered to 120 IOV customers with the aim of identifying those sub-populations producing more stable estimates of the social network size of the patients, following the procedure in Snidero *et al.* [27, 28]. After the analysis, 15 sub-populations were carefully selected. At this point, 5 more questions, representing the target sub-populations (*i.e.* people un-satisfied for on aspect or the other of health service) were added Table (**1**). These last 5 questions were replicating the main domains of the CRO questionnaire information: the distribution of signs in the Institute, personnel level of helpfulness and cooperation, medical personnel approach to health problems, short waiting time for medical consultation after scheduling an appointment, general feed-back on the service provided.

Summarizing, the final version of the questionnaire included: the number of people they know in 15 subpopulations of known size and the 5 questions related to PQC in the patients of IOV. More details on the NSUM procedure are provided in the Appendix as supplemental material.

### 2.3 Target Domains

More in detail, the target questions in the CRO questionnaire were: How do you evaluate directions and other information available for orientation in the Institute? How do you evaluate the cooperation and courtesy of the personnel working in the Institute? If you have ever been treated at this Institute, how do you evaluate the treatment received by doctors? How do you evaluate the waiting time for the medical consultation after scheduling an appointment?; How do you evaluate the general Service provided by the Institute? In the CRO questionnaire the 5 question were evaluate using a Likert scale from “good” to “inadequate”.

The NSUM questions for the target domain were constructed following the CRO target questions, but rephrasing them as: How do you evaluate directions and other information available for orientation in the Institute? How do you evaluate the cooperation and courtesy of the personnel working in the Institute? If you have ever been treated in the Institute, how do you evaluate the treatment received by our doctors? How do you evaluate the waiting time for the medical consultation after scheduling an appointment? How do you evaluate the general Service provided by the Institute? How many people who consider directions and other information available for orientation in this Institute inadequate do you know? How many people who consider the staff cooperation and courtesy inadequate in this Institute do you know? How many people dissatisfied with the treatment received by doctors operating in the Institute do you know? How many people who experienced very short waiting time (less than 10 days) for medical consultation after scheduling an appointment do you know? How many people dissatisfied with the general Services provided by this Institute do you know?

To compare CRO and NSUM estimates, CRO scores on the Likert scale were classified as “dissatisfied” if score was equal to 4, and as “satisfied” if lower than 4.

### 2.4 Statistical Methods

Statistical aspects related to the NSUM method are described in the Appendix. Questions were selected following the application of a linear model which associates the average number of people known by the 120 participants to the pilot survey to the total population size [24, 29]. Social network size estimates, as well as the estimates of the target population percentage sizes with their 95% confidence intervals were obtained applying the formulas provided in the Appendix for the NSUM.

A sensitivity analysis for the scale-up estimation was conducted through the leave-one out (LOO) technique, regularly reassessing and excluding data for each known size sub-population.

Analyses have been conducted using the R System [30].

## 3 RESULTS

Seven-hundred-eighty-three questionnaires were handed out. 521 were completed for the CRO and 341 for the NSUM, among which 42 were not included due to irrelevant or wrong answers. Only the 299 sets fully completed for both questionnaires were finally selected for the current analysis.

The distribution of some sample features is shown in Table **2**. The sample is prevalently composed by females and younger than the typical hospital care demography in Italy, with only 14% of the sample being older than 70 years.

Based on the first 15 questions (known size sub-populations) of the NSUM, we found out that the interviewees’ average social network size is estimated as 19.86 (95% C.I.19.62 - 20.10).

In this sense, a (severely) ill oncological patient has the capability of actively contacting about 20 people Using this estimated network size, the five target domains have been estimated Table (**3**). The dissatisfaction rates, as estimated with the NSUM approach, are ranging from 2.44% up to 6.82% for negative aspects. The question relative to a positive (short) waiting time report with the NSUM method reported a 3.85% of satisfaction. Table **3** also reports the analogous estimates as obtained with the traditional approach of direct questioning: dissatisfaction rates range from 0.17% up to 2.67%, the latter about the directions available in the hospital for patients. Noticeably, direct questioning about positive aspects (waiting time), reports a 36% of overall satisfaction.

To understand if and how estimates with the NSUM approach are depending on the particular choice of questions in the “known-size” part of the questionnaire, a sensitivity analysis to the choice of the known-size sub-populations on the estimates of the target domains has been performed. Estimates are presented in Table **4**, showing a very high degree of consistency and robustness to the choice of the know-size sub-populations.

Dissatisfaction rates, as estimated with the NSUM approach and stratified by age and gender are presented in Table **5**. In general, dissatisfaction rates are higher for females and for extreme ages (younger than 30 years and older than 60 years).

## 4 DISCUSSION

### 4.1 PQC Assessment

The issue of how direct the questions should be in order to catch a complex phenomenon like PQC has been widely debated: indeed, questionnaires asking for evaluations of health care in terms of satisfaction or dissatisfaction have been shown to be less discriminating than questionnaires that use terms such as good and bad or agree and disagree with concrete aspects of care [31, 32]. A discrepancy approach, both semantic and based on concrete situations, has been proposed, and some questionnaires measure preferences and experiences deriving evaluations from the two by calculating difference or ratio scores [33]. Although there is some evidence that patients are capable to distinguish between preferences and experiences, there is in general no validated framework for deriving evaluations from preferences and experiences [34-37]. Qualitative approaches have been proposed to examine patients’ experiences in more depth, in particular in some areas of specialty [38]. In general however, all such approaches use direct methods of interviewing [39].

For what concerns the general level of quality of services provided by hospitals, as emerged from a pan-European survey, the dissatisfaction rate is estimated ranging from 30% up to 45% [1]. Our current survey at IOV, conducted in a very specialized and quality-driven institute, and thus not directly comparable with such figures, is depicting a very different scenario in terms of PQC, being definitely very high according to both the CRO and the NSUM approach: overall, the percentage of dissatisfied users is respectively estimated as about 0.6% and 3.6%.

Research in the Italian context showed that satisfaction rates trespass the upper limit of 20% only in specific domains, whilst it ranges normally on the 5-8% range. In the oncological context, like the one in our study, 20% of patients were wanting improvement in aspects of care pertaining to doctors’ provision of information, *i.e.* ‘information on illness’, ‘information on resources for help’ (19%) and ‘information on medical tests’ (19%), whereas a lower proportion of patients wanted improvement in aspects of care relating to nurses’ availability (7%), nurses’ or doctors’ human quality (5 and 7%, respectively) and hospital comfortableness (4%) [40].

In our research, PQC is high for all domains related to professional aspects both for what concerns medical treatment (2.34%,95% C.I. 2.06-2.63%) and staff courtesy (2.4%, 95% C.I. 2.15-2.74%), in line with data coming from Europe, where satisfaction on medical and nursing staff is up to 70-80% [1]. In this context, nursing staff is widely recognized to play an essential role, representing often a gateway between the patient and the health care organization [41, 42]. Nurses interact with patients more often than any other health care professional in a hospital [43] and numerous study findings indicate that nursing care is a key determinant of overall patient satisfaction during a hospital admission [1, 44].

Nurse activities are moreover a key factor in providing complete, timely and understandable information about a patient’s illness and the development of therapy, which again is a pillar in PQC [45] along with other aspects like the time spent with the physician, the interpersonal skills of the physician, waiting time to get an appointment, empathy of personnel with the patient, the continuity of care provided [45]. Such aspects are not only a matter of self-consciousness, but are also widely discussed in the personal social network of the patients, as shown by the high degree of capability to respond to such questions indirectly as observed in the NSUM approach.

Structural aspects of the hospital are more critical and modifiable only on the long term but they might also be a matter of deeper discussion in the patient network. NSUM figures shows that 6.82% (95% C.I. 6.33-7.31%) of the patients are able to recognize among the persons connected with them an average number of 2-3 dissatisfied persons (out of a network size which on average accounts for about 20 people per patient). Furthermore, although not directly documented in the study, being the Oncological Institute a reference care provider for all over Italy, with patients coming also from southern Italian regions, a potential overestimation of the PQC using traditional methods is plausible and it is potentially attributable to cultural aspects and attitudes, which have been documented as influencing PQC in southern patients [46]. Noticeably, in particular with reference to the Italian context, a link between low PQC and compliance of patients to care has been documented, raising further concerns over the public health consequences of a potential under-estimation of dissatisfaction rates [47].

### 4.2 Applicability of NSUM

CRO-based estimates of the current level of satisfaction among patients are obtained using a classical approach, where people are directly asked about their preference and/or satisfaction regarding one or more services. This approach is known to be biased since the very early research on PQC assessment, in the sense that “*satisfaction scores may be falsely high, since most patients do not wish to give negative answers, or falsely low, since some patients are dissatisfied with life in general*” [48]. Nevertheless, the direct approach is still widely used and is a part of a routinely assessment in most Health Care facilities, both in Europe [14, 49] and in USA [50]. In the current survey, the dramatically low estimates of patients’ dissatisfaction rates as obtained with the CRO direct approach are most likely to be attributable to a bias in patients’ responses. Indeed, when asked indirectly with the NSUM approach, people provide figures on dissatisfaction rates which are 20-fold higher. This estimate is still lower than literature, but this might be attributable to the situation of a very well performing and good perceived service, which might be the case for a high specialty oncology hospital like IOV.

It is very difficult however to determine which can be, if any, the bias in the NSUM estimates. It is well reported in the technical literature about the potential barrier and transmission effects, which may cause people to under- or over-estimate the number of people known in each given situation [1, 51]. An indication on the magnitude of the bias could be provided, as recommended in Snidero *et. al* [29], by a sensitivity analysis of each target question to the choice of each specific sub-population used in getting the estimates of the social network size.

In this regard, we have noticed that outcomes are not particularly influenced by the target size population, except for the population number 13, whose influence is however not such high to change the overall conclusions of the research in terms of PQC at IOV.

What seems to matter most is the positive or negative meaning of target questions: indeed, the NSUM approach seems to underestimate “positive” qualities of the institute, showing for instance a very low degree of satisfaction rates about “short” waiting times “short waiting time for the medical consultation after scheduling an appointment”. This might be attributable to a low transmission of positive concepts among family members of peers within each person’s social network, suggesting that NSUM would be more appropriate for investigating negative or stigmatizing concepts more than positive ones. This should be the matter for further investigations.

In this sense, results of the NSUM are undoubtedly encouraging, although a more sensible and focused choice of subpopulations would be desirable for the future to enhance the quality of outcomes. As a critical point in the approach, it is worth noting that the number of questionnaires collected is below 50% of the total number handed out to participants. This may have been influenced by time constraints, unavoidable since the NSUM survey was forced to be conducted in conjunction with the CRO survey in a very tight time window of three days, for administrative and operational reasons. A wider survey, with more subpopulations and more time to be conducted would be helpful in providing a hopefully clearer picture of the applicability of NSUM in the context of PQC assessment.

There are other several measurable factors that influence the PQC in cancer patients, although many authors have also reported that customers satisfaction is largely influenced by the medical treatment received or the different cultural background, which leads patients to have different expectations on the “type” of treatment they hope to receive [52]. The NSUM estimates clearly show an age and gender effect on both the overall level of satisfaction and its specific professional and organizational aspects. Previous reports highlighted, in a different population, how younger ages are commonly associated with higher dissatisfaction rates [53]. In our analysis, NSUM estimates provide a different picture according to which aspect of PQC we focus on: an age effect in the sense of worse PQC for older people is characterizing the overall satisfaction and the organizational aspects, whereas an interesting U-shaped curve is derived for the courtesy, with youngest and oldest people being more un-satisfied than middle-aged patients. The left-part of the curve is in accordance with some UK studies, where higher degrees of un-satisfaction for communication, courtesy and in general emphatic matters are associated with age younger than 45 years [54]. Noticeably, dissatisfaction rates are higher for females than for males, in particular for what concerns organizational aspects like indications and directions within IOV and overall attitudes toward IOV. This is in accordance with a large survey focused on recommendations of the health care facility to others, where females were less prone to recommend the hospital to others [18].

## CONCLUSION

Well known limits of the classical, direct techniques to interview people to estimate PQC are clearly emerging from our CRO survey. The NSUM alternative approach, based on the concept of social network, is promising and deserves further investigations and fine tuning.

## Figures and Tables

**Fig. (A1):**
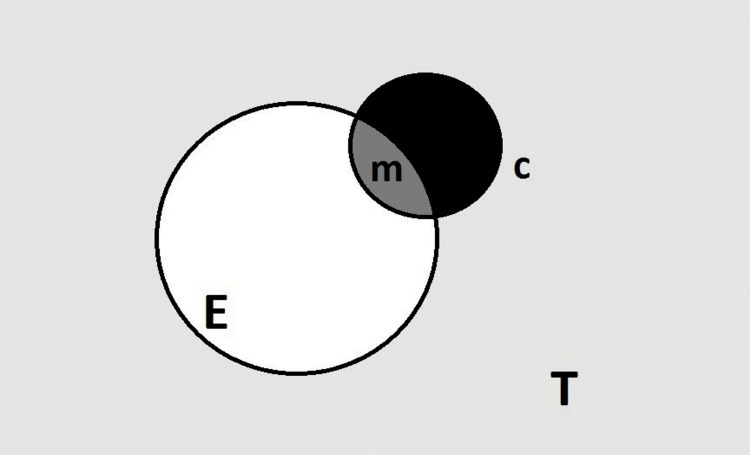
Populations of target size selected for the scale-up questionnaire.

**Table 1:** Fifteen populations of known size selected for the first part of the PQC-NSUM questionnaire [26].

S.No	**Population of Known Size Selected**	**Population Size**
**1**	People who separated in 2010	7,079
**2**	Foreign residents	366,847
**3**	People with at least one foreign parent	8,410
**4**	Victims of car accidents with injuries	15,564
**6**	University Professors	5,077
**7**	Unemployed people in the region	2,493,613
**8**	People living in a 150 m2 house	2,100,510
**9**	People living in a 5-room house	7,079
**10**	People working part-time	239,154
**11**	3-member families	1,296,521
**13**	People who married in 2010	17,474
**14**	Children born in 2010	42,604
**15**	People above 14 with smoking habit	13,519,697
**24**	People above 3 who declare to train for a sport regularly	1,065,725
**28**	People who walk to work	574,226

**Table 2:** Demographic characteristics of patients (n= 299) (N= Absolute Number; NA= not responders)

**Characteristics of Patients**	**N**	**%**
Gender		
Female	226	75.3
Male	70	23.3
NA	4	1.3
Age		
Up to 18	5	1.6
from 19 to 30	16	5.3
from 31 to 50	99	33.0
from 51 to 60	76	25.3
from 61 to 70	59	19.6
over 71	43	14.3
NA	2	0.6
Institute choice		
Independently	87	29.0
GP	43	14.3
Specialist doctor	118	39.3
Other	43	14.3
NA	9	3.0

**Table 3:** Estimates of PQC of the population target size under study, on a regional basis, obtained on the basis of the 5 target domains with the NSUM approach and with the traditional CRO approach. Percentage (%) and absolute numbers (N) for the CRO approach.

***PQC domains***	***%***	***IC (%)***
NSUM Approach		
Inadequate directions	6.82	6.33 - 7.31
Inadequate cooperation and courtesy of the personnel working in the Institute	2.44	2.15 - 2.74
Inadequate treatment received by Doctors of the Institute	2.34	2.06 - 2.63
Short waiting time for the medical consultation after scheduling an appointment	3.85	3.49 - 4.22
Inadequate general Service provided by this Institute	4.28	3.89 - 4.67
**CRO Approach**		
Inadequate directions	2.67 (8)	0.84-4.49
Inadequate cooperation and courtesy of the personnel working in the Institute	0.33 (1)	0.00–0.99
Inadequate treatment received by doctors of the Institute	0.17 (1)	0.00-0.63
Short waiting time for the medical consultation after scheduling an appointment	36.00 (108)	30.57-41.43
Inadequate general Service provided by this Institute	0.67 (2)	0.00-1.59

**Table 4:** The sensitivity analysis of percentage (%) and the mean social network sizes estimates with NSUM, performed using the Leaving One Out (LOO) approach.

**Population of known Size Eliminated from The Analysis**	**Inadequate Directions**			**Inadequate Cooperation and Courtesy of the Personnel**			**Inadequate Treatment Received by Doctors**			**Short Waiting time for the Medical Consultation after Scheduling an Appointment**			**Inadequate General Service Provided**			**Social Network**		
																**Estimate**		
	**%**	**95%**	**C.I.%**	**%**	**95%**	**C.I.%**	**%**	**95%**	**C.I.%**	**%**	**95%**	**C.I.%**	**%**	**95%**	**C.I.%**	**%**	**95%**	**C.I.%**
People who separated in 2010	6.98	6.3	7.66	2.5	2.09	2.91	2.4	2	2.8	3.94	3.43	4.45	4.38	3.84	4.92	8.81	6.06	11.55
Foreign residents	7.45	6.73	8.18	2.67	2.23	3.11	2.56	2.13	2.99	4.21	3.66	4.76	4.67	4.1	5.25	7.41	4.89	9.92
People with at least one foreign parent	7.13	6.43	7.83	2.55	2.14	2.97	2.45	2.04	2.86	4.03	3.5	4.55	4.47	3.92	5.02	8.84	6.09	11.59
Victims of car accidents with injuries	6.97	6.29	7.65	2.5	2.09	2.9	2.39	1.99	2.79	3.93	3.42	4.45	4.37	3.83	4.91	6.53	4.17	8.89
University Professors	7.21	6.51	7.92	2.58	2.16	3.01	2.48	2.06	2.89	4.07	3.54	4.6	4.52	3.96	5.08	7.78	5.2	10.16
Unemployed people in the region	7.88	7.11	8.65	2.82	2.36	3.28	2.7	2.25	3.16	4.45	3.87	5.03	4.94	4.33	5.55	7.61	5.06	10.16
People living in a 150 m2 house	6.67	6.01	7.32	2.39	2	2.78	2.29	1.91	2.67	3.76	3.27	4.25	4.18	3.66	4.69	9.63	6.76	12.5
People living in a 5-room house	6.61	5.96	7.26	2.37	1.98	2.75	2.27	1.89	2.65	3.73	3.25	4.22	4.14	3.63	4.66	10.44	7.45	13.43
People working part-time	7.22	6.52	7.93	2.59	2.17	3.01	2.48	2.07	2.89	4.08	3.55	4.61	4.53	3.97	5.09	9.35	6.53	12.18
3-member families	7.15	6	7.84	2.56	2.14	2.98	2.45	2.04	2.86	4.03	3.51	4.56	4.48	3.72	5.03	9.83	6.93	12.07
People who married in 2010	6.92	6.45	7.59	2.48	2.07	2.88	2.37	1.98	2.77	3.91	3.4	4.41	4.34	3.8	4.87	9.26	6.44	12.07
Children born in 2010	7	6.24	7.69	2.51	2.1	2.92	2.4	2	2.81	3.95	3.44	4.47	4.39	3.85	4.93	9.27	6.45	12.08
People above 14 with smoking habit	2.98	6.32	3.27	1.07	0.89	1.24	1.02	0.85	1.19	1.68	1.46	1.9	1.87	1.64	2.1	24.24	19.69	28.79
People above 3 who declare to train for a sport regularly	7.28	2.69	7.99	2.61	2.18	3.03	2.5	1.94	2.71	4.11	3.33	4.64	4.56	4	5.12	9.73	6.84	12.61
People who walk to work	6.77	6.11	7.43	2.42	2.03	2.82	2.32	2.08	2.91	3.82	3.57	4.32	4.24	3.93	4.77	9.5	6.65	12.35

**Table 5:** Target population size estimates stratified by age and gender classes.

	**up to 30**			**from 31 to 60**			**over 61**			**Male**			**Female**		
	**%**	**95% C.I.**		**%**	**95% C.I.**		**%**	**95% C.I.**		**%**	**95% C.I.**		**%**	**95% C.I.**	
Inadequate directions	0.51	0.00	1.21	4.09	3.62	4.55	3.18	2.57	3.79	1.98	0.61	0.94	4.36	3.96	4.75
Inadequate cooperation of personnel working in the Institute	2.79	1.14	4.44	0.73	0.53	0.93	2.45	1.91	2.98	1.01	0.82	1.19	1.46	1.23	1.69
Inadequate treatment received by Doctors of the Institute	0.76	0.00	1.62	0.55	0.38	0.72	2.90	2.32	3.49	0.78	0.61	0.94	1.31	1.10	1.53
Short waiting time for the medical consultation after scheduling an appointment	1.78	0.46	3.09	2.04	1.71	2.37	2.17	1.67	2.68	1.38	1.16	1.60	2.44	2.15	2.74
Inadequate general service provided by this Institute	3.05	1.32	4.77	0.72	0.52	0.91	5.75	4.93	6.57	0.78	0.61	0.94	3.02	2.69	3.34

**Table A1:** Populations of known size published on the Central Statistics Institute website used for the pilot questionnaire [26].

	**Population of Known Size**	**Population Size**
**1**	People who separated in 2010	88191
**2**	Foreign residents	4570317
**3**	People with at least one foreign parent	104773
**4**	Victims of car accidents with injuries	205638
**5**	People who graduated in 2008	20204
**6**	University Professors	63249
**7**	Unemployed people in the region	112000
**8**	People living in a 150 m2 house	1953963
**9**	People living in a 5-room house	2319640
**10**	People working part-time	222469
**11**	3-member families	1206066
**12**	Cohabiting couples	43779
**13**	People who married in 2010	217700
**14**	Children born in 2010	530770
**15**	People above 14 with smoking habits	13519697
**16**	Women who miscarried in 2006	74117
**17**	People using the mass media (newspapers, magazines, TV, radio...)	35757801
**18**	6-year old children who have been to the theatre in the last year	13277191
**19**	6-year olds and above who attended discos, night clubs, pubs or other dancing clubs at least once in 2009	13570185
**20**	6-year olds and above who read a book in 2011	27463778
**21**	3-year olds and above who used a PC and Internet in 2011	31647003
**22**	People working in public libraries (Veneto)	135
**23**	People who have borrowed a book from a public library (Veneto)	14942
**24**	People above 3 who declare to train for a sport regularly	32844653
**25**	People who have few or many problems to reach the emergency room	13277191
**26**	14-year olds and above who use means of transportation at least few times a year	14974731
**27**	People who attend places of worship	19308905
**28**	People who walk to work	7153920
**29**	People who go to school by bus	7153920

## LIST OF ABBREVIATIONS

(PQC): = Perceived quality of health care services
(CRO): = Customer Relations Office
(NSUM): = Network Scale-Up Method
(IOV): = Oncology Hospital in Veneto
(CUP): = Center Unique Reservation

## APPENDIX A

The NSUM is a social network estimator for the size of hidden and hard-to-count subpopulations. The main concept of social network can be expressed as the set of people known by each person. Starting from a subpopulation *E* of the full population *T*, this method is based on the assumption that the ratio between the unknown size *e* of *E* and the size *t* of *T* is proportional to the ratio of the mean numbers of people in *E* and in *T* known by the respondent (Fig. **A1**). On the other hand, if *c* is the number of persons known by each respondent and *m* is the mean number of persons known by each respondent in the subpopulation *E*, this method assumes that *m/c = e/t*.

In this paper we used a maximum likelihood estimator [55] that relies on the assumption that the number *m_ij_*, of each target population, known by the *i* - th respondent, follows a Binomial distribution:

(1) $$\mathrm{Prob}\left(m_{\mathrm{ij}}\right)=\left(\overset{c_{i}}{\underset{m_{\mathrm{ij}}}{}}\right)p^{\mathrm{mij}}{\left(1-p_{j}\right)}^{c_{i}-m_{\mathrm{ij}}}$$

(2) $$p_{j}=\frac{e_{0}}{t}$$

Where *c_i_* is the social network size of *i* - th respondent, and *e_j_* is the unknown size of the *j* - th target population.

The maximum likelihood scale-up estimator for the size *e_j_* of the target subpopulation *E_j_* is given by

(3) $$\hat{e_{j}}=t\frac{\sum_{i}m_{\mathrm{ij}}}{\sum_{i}c_{i}}$$

where the size of the general population *T* is multiplied by the ratio between the total number of the *j* - th target population known by the respondents and the sum of the respondent social network sizes. It has been proven that equation (3) is an unbiased estimator [19, 21].

The estimator (3) requires just computing the sum of the social network’s size *c_i_* over all respondents. These latter parameters were estimated in this work by using the proportional estimator [29]. This estimator relies on the same basic underlying idea of the scale-up method: to estimate the social network size of each respondent. People are asked about how many people they know in several subpopulations of known size. Therefore, the proportional estimator for the social network size of the *i* - th respondent is given by the following formula:

(4) $$\hat{c_{j}}=t\frac{\sum_{k=1}^{L}m_{\mathrm{ik}}}{\sum_{k=1}^{L}e_{k}}$$

Where *m_ik_* is the number of people known by the *i* - th respondent in the subpopulation of known size . As in *e_k_* the scale-up estimator the quantities cj are assumed known, in this case they are estimated from subpopulations of known size (*i.e.*, the *e_k_* terms are fixed).
